# Iodine status of pregnant women from the Republic of Cyprus

**DOI:** 10.1017/S0007114522000617

**Published:** 2023-01-14

**Authors:** Andrea Cannas, Margaret P. Rayman, Ourania Kolokotroni, Sarah C. Bath

**Affiliations:** 1 Department of Nutritional Sciences, Faculty of Health and Medical Sciences, University of Surrey, Guildford, Surrey, GU2 7XH, UK; 2 Department of Primary Care and Population Health, University of Nicosia, Medical School, Nicosia, 1700, Cyprus

**Keywords:** Iodine, Iodine deficiency, Iodine intake, Iodine status, Republic of Cyprus, Pregnancy, Supplement

## Abstract

Iodine supply is crucial during pregnancy to ensure that the proper thyroid function of mother and baby support fetal brain development. Little is known about iodine status or its dietary determinants in pregnant women in the Republic of Cyprus. We therefore recruited 128 pregnant women at their first-trimester ultrasound scan to a cross-sectional study. We collected spot-urine samples for the measurement of urinary iodine concentration (UIC, µg/l) and creatinine concentration (Creat, g/l), the latter of which allows us to correct for urine dilution and to compute the iodine-to-creatinine ratio (UI/Creat). Women completed a FFQ and a general questionnaire. We used a General Linear model to explore associations between maternal and dietary characteristics with UI/Creat. The median UIC (105 µg/l) indicated iodine deficiency according to the WHO criterion (threshold for adequacy = 150 µg/l), and the UI/Creat was also low at 107 µg/g. Only 32 % (*n* 45) of women reported the use of iodine-containing supplements; users had a higher UI/Creat than non-users (131 µg/g *v.* 118 µg/g), though this difference was NS in the adjusted analysis (*P* = 0·37). Of the dietary components, only egg intake was significantly associated with a higher UI/Creat in adjusted analyses (*P* = 0·018); there was no significant association with milk, dairy products or fish intake. Our results suggest that pregnant women in Cyprus have inadequate iodine status and are at risk of mild-to-moderate iodine deficiency. Further research on dietary sources in this population is required.

Inadequate iodine status negatively affects thyroid hormone synthesis^(1)^ and may impair brain development in fetal life, particularly up until the start of the second trimester^(2,3)^. Severe iodine deficiency during pregnancy has considerable consequences for the developing child^(4,5)^. However, the incidence of severe iodine deficiency has been reduced in many countries owing to the provision of iodised salt^(6)^. The current focus of research is on the rising prevalence of mild-to-moderate iodine deficiency in at-risk-populations, particularly pregnant women, and those of reproductive age^(6)^. The accumulating evidence from observational cohort studies has shown an association between mild-to-moderate ID in pregnancy and poorer offspring neurodevelopment outcomes, lower child IQ^(2,3)^, spelling scores^(7)^, language skills^(8)^ and psychomotor development^(9,10)^. The negative effects of iodine deficiency during pregnancy may not be completely ameliorated by iodine sufficiency during childhood and some effects may persist^(7,11,12)^, suggesting that iodine deficiency during pregnancy may have long-lasting implications for the child.

The WHO recommendation for iodine intake in pregnancy and lactation is 250 µg/d, which is considerably higher than the 150 µg/d recommendation for adults^(13)^. To assess population iodine status, the WHO recommends collecting spot-urine samples for the measurement of urinary iodine concentration (UIC) and comparing the median UIC to the WHO cut-off for iodine adequacy^(14)^. Population iodine deficiency is noted if the median is <100 µg/l in adults or school-aged children or <150 µg/l for pregnant women.

Mild-to-moderate iodine deficiency in pregnancy exists in many countries^(6)^, particularly in Europe^(15,16)^. Iodine status in the general population in Cyprus was thought to be sufficient (median UIC 120 µg/l)^(17)^, but this classification is based on data from school-aged children from Northern Cyprus from a national survey that is more than 20 years old^(17,18)^. There is just one study of iodine status of pregnant women in Cyprus and that is from a study of 258 women, also from Northern Cyprus, which found the median UIC to be low, at 110 µg/l^(19)^. The location of the studies is important as Cyprus has been divided into the Republic of Cyprus (populated by Greek-Cypriots) and the self-declared northern part of the island (populated by Turkish-Cypriots) since 1974. There may be differences between the two regions of Cyprus as the northern part of the country may be exposed to additional iodine because of reliance on food imports from Turkey, where there is mandatory salt-iodisation^(20)^, and this may not be the case in the Republic of Cyprus.

There is evidence to suggest a marked transition from the traditional Mediterranean diet, which is rich in iodine-containing foods, to a more Westernised dietary pattern in Cypriot children, young adults^(21,22)^ and young women^(23)^. It is known that goat and sheep milk is more iodine-rich than cows’ milk^(24,25)^, and this is relevant in Cyprus, which is heavily reliant on local goat dairy produce. In Cyprus, there is no official recommendation for pregnant women to take an iodine supplement, and it is uncertain whether women are aware of the increased iodine requirements in pregnancy, or the dietary sources of iodine.

Iodine status is very important in early pregnancy^(19)^, but there are limited data on this time period in Cyprus as the previous study of iodine status in Turkish-Cypriot pregnant women had a relatively small number of samples from the first trimester (*n* 34, 13 % of total in the study). There are also no data on the intake of iodine-rich foods or the use of iodine-containing supplements in pregnancy in Cyprus, which are needed in order to identify population groups who may be at risk of deficiency. Our study aim was therefore to assess iodine status in a group of Greek-Cypriot pregnant women in the first trimester and to assess dietary iodine intake and use of iodine-containing supplements. We hypothesised that women would be iodine deficient by the WHO criterion, and that milk and dairy products (particularly local goat dairy products) would be positively associated with iodine status.

## Experimental methods

### Study recruitment

Between May 2019 and June 2019, healthy pregnant women were recruited at their first trimester visit for an ultrasound scan (which takes place between 10 and 13 weeks of pregnancy) to our cross-sectional study. Recruitment took place at the private outpatient obstetric AAK Ultrasound and Foetal Medicine Centre in the capital of Cyprus, Nicosia. The participants were asked to provide a spot-urine sample after their appointment and complete both a general questionnaire and a short iodine-specific FFQ. Clinics were conducted both in the morning and in the afternoon. We aimed to collect a minimum of 125 urine samples as that has previously been estimated to give population iodine status with 95 % confidence and a precision range of ±10 %^(26)^.

We included healthy adult pregnant women, and excluded those who had pre-existing thyroid disease, used thyroid medication (e.g. levothyroxine, carbimazole) and had *in vitro* fertilisation or other assisted reproductive techniques in current or past pregnancies. Participants were excluded if they were < 18 years of age.

This study was conducted according to the guidelines laid down in the Declaration of Helsinki, and procedures involving human subjects were approved by the Bioethics Cyprus Committee, Nicosia (EEBK/EΠ /2019/26) and the Faculty of Health and Medical Sciences Ethics Committee at the University of Surrey, Guildford (FER-1819-042). Written informed consent was obtained from all subjects. Approval was also obtained from Data Protection officers following submission of signed Standard Contractual Clauses between countries.

### Spot urine collection and laboratory analysis

Spot-urine samples were collected from each participant during the clinic (at any time during the day) and stored at -20°C at the University of Nicosia until recruitment was complete. Samples were shipped on dry ice to the laboratory for iodine and creatinine analysis. Analysis took place at the Trace Element Unit, Southampton General Hospital, Southampton, UK. Urinary creatinine was determined by the UniCel DxC Synchron Clinical System Analyzer (Beckman Coulter) by the Jaffe rate method. UIC was measured by using a dynamic reaction-cell inductively-coupled-plasma mass spectrometer (Sciex Perkin-Elmer). The measurement method used has been reported previously^(27)^. We verified the accuracy of the method with the certified reference materials, Seronorm Trace Elements urine Levels 1 and 2 (Nycomed Pharma, Norway). Our observed mean values for the certified reference materials (L1A, L2A) and their diluted (1/2) counterparts (L1B, L2B) were 111·4 µg/l (sd 8·9, *n* 11) for L1A (certified mean 105·41 µg/l, range 84–126); 51·6 µg/l (sd 2·4, *n* 11) for L1B (certified mean 53·34 µg/l, range 42–63·5); 306·2 µg/l (sd 8·1, *n* 9) for L2A (certified mean 292·1 µg/l, range 241–343), and 164 µg/l (sd 5·4, *n* 9) for L2B (certified mean 146·1 µg/l, range 121–178). The laboratory participates in the Quebec Interlaboratory Comparison Program and eight External Quality Assurance (EQA) control samples were used for urinary iodine concentration; the EQA values were within consensus defined in the multi-laboratory programme, with bias of -2·3–1 %, demonstrating acceptable analytical accuracy. Within-run precision gave a relative standard deviation of 3·3 % at 53 µg/l, 4·9 % at 105 µg/l, 2·8 % at 146 µg/l and 2·5 % at 292 µg/l. Between-run precision was 4·2 % relative standard deviation at 53 µg/l, 6·7 % at 105 µg/l, 1·7 % at 146 µg/l and 1·6 % at 292 µg/l.

### Demographic, anthropometric and health-related data

Women were given a general questionnaire to complete to provide data on age, self-reported weight and height, smoking status and information on dietary preferences (i.e. vegan or vegetarian), food intolerances or allergies. Women were asked to select their age group, from six age brackets (range from 18–24 years to >45 years); the age groups were collapsed into three categories for the purposes of analysis: 18–29, 30–34 and 35–44 years (none of the women were >45 years). BMI was calculated from the height and weight data and, for the purposes of statistical analysis, was categorised as <25 or ≥25 kg/m^2^.

### Dietary assessment and analysis

Consumption of iodine-rich foods was assessed by an eighteen-item FFQ that was designed to capture the frequency of intake of iodine-rich food, but not to estimate total iodine intake. The FFQ was based on one that had previously been validated in the EPIC-Norfolk study^(28)^ and which had been used in UK iodine research^(29)^; it was modified to reflect local iodine-rich foods and customs in the Republic of Cyprus (e.g. to include traditional cheeses such as halloumi or feta). The FFQ asked about intake of seafood (white fish, oily fish and shellfish), meat and poultry, dairy products (cheese, cream, Greek yoghurt, low fat yoghurt, butter and dairy desserts) including traditional cheeses (halloumi, feta and anari cheeses) and other traditional dairy products (cheesepie, trahanas soup), and iodised salt. One of the authors was present during the completion of the FFQ, which gave an opportunity for clarification of ‘iodised salt’.

The FFQ assessed daily use of kelp/seaweed supplements and multimineral-vitamin supplements (with brand names provided by participants). We calculated the dose of iodine, if any, by investigating supplement brands reported. Participants were grouped into iodine-containing supplement users and non-users. The participants who did not specify the supplement brand on the FFQ were labelled as ‘not known’ and were excluded when comparing iodine status between groups.

The FFQ asked participants to record the type of milk consumed (cow, goat, soya or other plant-based milk alternatives). Milk consumption was grouped into four categories (none, <140, 140–280, >280 ml/d; on the FFQ, the units for milk volume were given as both ml and cups) and for the purposes of examining the relationship with iodine status, only those who consumed cow or goat milk were included (as milk alternatives are generally a poor source of iodine^(30)^). Those who selected two options (*n* 7) were excluded from the analyses.

Weekly egg consumption was assessed by six options ranging from ‘none’ to ‘more than 4/week’. As a result of low numbers in some categories, for the purposes of statistical analysis, egg intake was grouped as (i) none; (ii) 1–3 and (iii) 4 or more eggs/week.

There were seven frequency options for all other foods and frequency options of individual items of dairy, meat and seafood were converted to weekly portions according to the following formula (as in previous research^(29)^): never/rarely = 0, once in 2 weeks = 0·5, once a week = 1, 2–3 times/week = 2·5, 4–6 times/week = 5, once a day = 7 and more than once a day = 10. The portions of each dairy, meat or seafood item were summed and grouped into broader food groups: (i) seafood (shellfish, white fish and oily fish); (ii) meat and poultry (meat, poultry) and (iii) dairy products (cream, Greek yoghurt, low fat yoghurt, dairy desserts, cheese, traditional dairy cheeses, butter and other traditional products).

For the purposes of statistical analysis, the broad food groups were grouped into three categories. Intake of dairy products was grouped as (i) up to 10 portions/week; (ii) >10 and ≤18 portions/week and (iii) more than 18 portions/week; meat and poultry intake was grouped as (i) up to 3·5 portions/week; (ii) >3·5 and ≤ 5 portions/week and (iii) more than 5 portions/week. Due to the overall low consumption of seafood, intake was dichotomised as (i) less than a portion/week or (ii) one portion/week or more.

### Classification of iodine status and estimated iodine intake

The median UIC was used to classify the population group by the WHO criterion for iodine adequacy, where a median UIC < 150 µg/l indicates iodine deficiency in the group^(14)^. We do not report the percentage below the threshold for adequacy as UIC data cannot be used in this way^(31)^. UIC values were expressed in relation to creatinine excretion, to give the iodine-to-creatinine ratio (UI/Creat; µg/g), which corrects for the urine dilution. The UI/Creat is a more appropriate proxy for individual status, especially in groups of the same sex and of similar age (as is the case in our study)^(32,33,34)^. When exploring the associations between diet (from the FFQ) and participant characteristics (e.g. age), we used UI/Creat.

To estimate the prevalence of iodine deficiency in this cohort of pregnant women, we applied the estimated average requirement (EAR) cut-point method^(35)^. We previously estimated that the EAR cut-point for 24-h urinary iodine excretion in pregnancy is 160 µg/24 h^(27,29)^. To estimate 24-h urinary excretion we multiplied the UI/Creat by 1·23, which is the expected daily excretion of creatinine (g/d) in women aged 18–43 years^(32)^.

### Statistical analysis

UIC (µg/l), UI/Creat (µg/g) and urine iodine excretion (µg/d) were not normally distributed and therefore we report the median and the 25^th^ and 75^th^ percentiles. UI/Creat was transformed using the natural logarithm, which enabled the use of parametric tests. Independent *t*-tests or one-way ANOVA was used to compare (log-transformed) UI/Creat between groups in univariate analyses. A General Linear Model was constructed to adjust the analysis by those factors that were associated with iodine status in univariate analysis (using variables with *P* < 0·2).

Significance was set at *P* < 0·05, unless otherwise specified. All analyses were performed using the Statistical Package for Social Sciences (Version 27; IBM SPSS, Inc.).

## Results

**Fig. 1.**
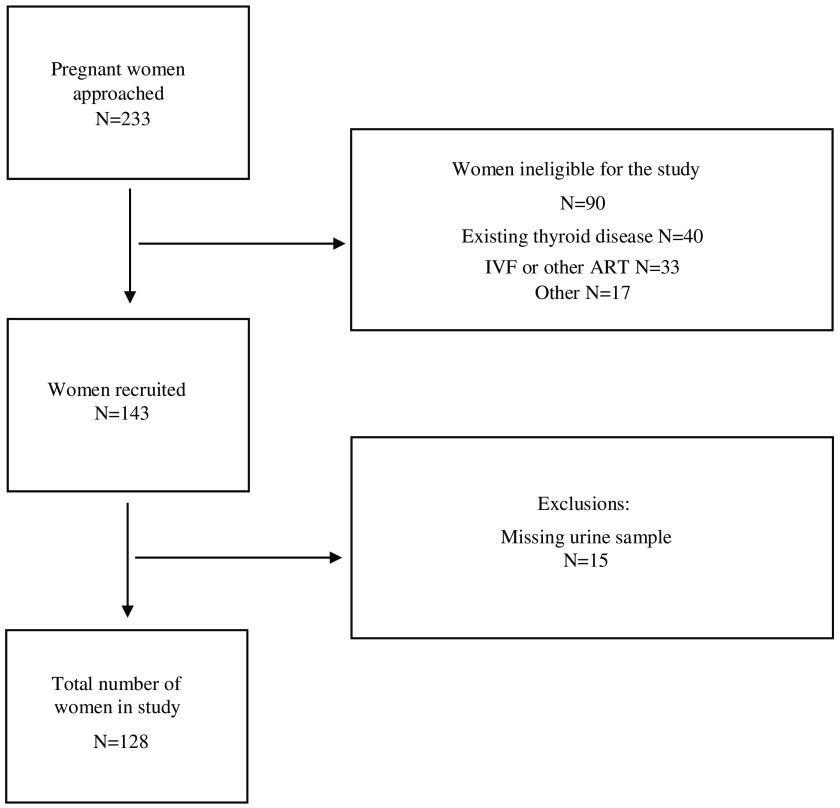
Flow diagram of recruitment into the study. IVF, *in vitro* fertilisation; ART, assisted reproductive techniques.

Of a total of 233 women were approached for participation in the study, 143 (61 %) were eligible and provided informed consent. Urine samples were not provided by fifteen women, leaving a total of 128 women in the study with both a urine sample and questionnaire data (Fig. 1).

The median BMI was 22·9 kg/m^2^ (range: 17–39 kg/m^2^), and most women were in the 30–34 years age category. In total, 121 (95 %) women were omnivores, three (2·3 %) were pescatarians and four were vegetarians or vegans (3·1 %). Most participants (*n* 111, 87 %) did not report allergy/intolerances but dairy, egg or other food allergy/intolerance was reported by *n* 11, *n* 2 and *n* 4 participants, respectively.

### Iodine status and dietary iodine intake in relation to dietary guidelines

The median UIC (mUIC) and UI/Creat values are summarised in Table 1. The mUIC was 105 µg/l, classifying this group of pregnant women as mildly-to-moderately iodine deficient by the WHO threshold (‘inadequate’ if mUIC < 150 µg/l)^(14)^. Twenty-four percent (*n* 31) had a UIC < 50 µg/l and three women (2·3 %) had a UIC ≥ 500 µg/l, two of whom were taking iodine-containing supplements. Even after accounting for urine dilution by using UI/Creat, the median was low at 107 µg/g and suggestive of iodine deficiency in the cohort. Using the EAR cut-point method, 66·4 % (*n* 85) had an estimated 24-h urine iodine excretion below the EAR (160 µg/24 h).

**Table 1.** Urinary iodine status in first-trimester pregnant women in the Republic of Cyprus (*n* 128) reported as urinary iodine concentration (UIC) (µg/L), urinary iodine-to-creatinine ratio (UI/Creat) (µg/g) and estimated 24-h urinary iodine excretion (UIE) (µg/d) (Median values and percentiles)

	Median	25th–75th percentile
Urinary iodine concentration (UIC) (µg/l)	105	51·2–160
Urinary iodine-to-creatinine ratio (UI/Creat) (µg/g)	107	75–163
Estimated 24-h Urinary Iodine Excretion (UIE) (µg/d)*	132	92–200

* Age- and sex-adjusted iodine-to-creatinine ratio calculated on the basis of an expected daily creatinine excretion of 1.23 g (Knudsen 2000).

### Demographic and dietary predictors of iodine status

Iodine status was not significantly different by maternal age (*P* = 0·62), smoking status (*P* = 0·85) or maternal BMI (*P* = 0·18; Table 2).

**Table 2.** Univariate comparisons of iodine-to-creatinine ratio (UI/Creat) (µg/g) according to participant characteristics and dietary intake (estimated from FFQ) (Number and percentages; median values and percentiles)

	*n*	%	Iodine-to-creatinine ratio (µg/g)		*P* †
			Median	25^th^–75^th^ percentile	
Age group (years)					0·62
18–29	36	28	100	73–132	
30–34	56	44	120	74–195	
35–44	36	28	103	79–156	
BMI category (kg/m^2^)					0·18
<25	89	70	107	78–179	
≥25·0	39	30	104	74–138	
Smoking status					0·85
Ex-smoker	45	35	99	75–141	
Non-smoker	72	56	106	72–173	
Smoker	11	9	124	111–163	
Iodised salt use					0·21
Non-consumer	115	90	128	91–200	
Consumer	13	10	162	117–206	
Kelp and/or iodine-containing supplements*					0·04
Non-consumer	74	61	91	67–143	
Consumer	45	32	110	92–183	
Daily milk volume (cows’/goats’ only)					0·65
None	13	10	105	81–157	
<140 ml	45	35	107	80–138	
140–280 ml	18	14	98	70–144	
>280 ml	27	21	102	63–187	
Dairy products – weekly intake of portions of all dairy products except milk					0·06
Up to 10/week	25	19	90	61–129	
>10 and ≤ 18/week	42	33	97	78–152	
>18 week	61	48	123	96–192	
Eggs – weekly intake					0·015
None	43	34	84	62–142	
One to three	44	34	104	75–143	
Four or more	41	32	183	109–440	
Seafood – weekly intake of portions of white, oily and shellfish					0·08
<1 portion/week	50	39	95	67–138	
1 portion or more	78	61	108	83–173	
Meat and poultry – number of portions/week					0·39
Up to 3·5/week	59	46	102	63–139	
Between 3·5 and 5/week	44	34	107	79–193	
More than 5/week	24	20	109	85–142	

N/A, variable not entered into the multivariate analyses.

* Missing data for nine subjects who did not know iodine content of supplement.

† *P* value for comparison between categories from independent *t* test or ANOVA, conducted on log-transformed iodine-to-creatinine values.

Use of a multivitamin-mineral supplement was reported by 59 % (*n* 75) of the group, but only 32 % (*n* 45) were using an iodine-containing supplement. Supplement brand names were reported by sixty-four participants (50 %), so we were able to identify that the dose of iodine ranged from 75 to 200 µg/d, with a median iodine content of 150 µg. UI/Creat was higher in iodine-supplement users than non-users, which was significant in the univariate analysis (Table 2), but not after adjusting for other factors (Table 3).

**Table 3.** Adjusted predictors of iodine-to-creatinine ratio (UI/Creat) (µg/g) from a general linear regression model (Number and percentages; mean values and 95 % confidence intervals)

	*n*	%	Iodine-to-creatinine ratio (UI/Creat) (µg/g)		Adjusted *P* †
			Geometric mean*	95 % CI	
BMI category (kg/m^2^)					0·50
< 25	89	70	130	111, 152	
≥25·0	39	30	120	96, 150	
Kelp and/or iodine-containing supplements					0·37
Non-consumer	74	61	118	98, 141	
Consumer	45	32	131	107, 161	
Dairy products – weekly intake of portions of all dairy products except milk					0·16
Up to 10/week	25	19	109	88, 136	
>10 and ≤ 18/week	42	33	124	100, 155	
>18 week	61	48	143	114, 179	
Eggs – weekly intake					0·018
None	43	34	103	79, 134	
One to three	44	34	105	92, 120	
Four or more	41	32	179	126, 254	
Seafood – weekly intake of portions of white fish, oily fish and shellfish					0·31
<1 portion/week	50	39	118	96, 143	
1 portion or more	78	61	132	110, 159	

* Analysis conducted on log-transformed iodine-to-creatinine values. Geometric mean calculated through back-transformation of log values.

† Variables identified from univariate analyses were entered into the model if *P* < 0·2, i.e. BMI, kelp and/or iodine-containing supplements, dairy products, eggs and seafood.

Model *R* ^2^ = 0·153.

The majority of women did not use iodised salt (*n* 115; 90 %), and there was no difference in UI/Creat between consumers and non-consumers (*P =* 0·21; Table 2).

The most commonly consumed type of milk was cows’ milk (*n* 93; 73 %) followed by goats’ milk (*n* 10; 8 %); soya or other plant-based milk alternatives were used by eighteen participants (*n* 18; 14 %). There was no significant difference in UI/Creat according to the intake of milk (*P* = 0·65) (restricted to cows’/goats’ milk; Table 2). Dairy-product intake was significantly positively associated with UI/Creat in univariate analysis (Table 2) but was not significant in the multivariate analysis (*P* = 0·16). When examining the individual components of the dairy category, the strongest predictor, and the only significant item to relate to UI/Creat, was the traditional cheese category (data not shown).

When combined into a seafood category, 61 % of women reported consuming at least one portion of seafood per week. While there was a positive relationship between seafood consumption and UI/Creat, the difference was not statistically significant (Tables 2 and 3). A proportion of pregnant women were non-consumers of different seafood types: 36 % (*n* 46) were non-consumers of white fish, 42 % (*n* 54) did not consume oily fish and 61 % (*n* 78) were non-consumers of shellfish.

In the multivariate analysis, the only factor that was significantly related to UI/Creat was the intake of eggs (positive association). The final model explained 15·3 % of the variance in the iodine-to-creatinine ratio (adjusted *R* ^2^ = 0·153).

## Discussion

This was the first cross-sectional national study carried out in the Republic of Cyprus that assessed iodine status in first-trimester pregnant women and explored their dietary iodine intake. The results support our original hypothesis as we found that the pregnant women were iodine deficient according to the WHO criterion^(14)^; the median UIC of the women in our study (mUIC 105 µg/l) was below the 150 µg/l cut-off that is used by WHO to indicate iodine deficiency and suggests mild-to-moderate deficiency. Our median UIC value was similar to that of women in the first trimester from the previous study in Northern Cyprus (108 µg/l)^(19)^. Taken together, the results suggest insufficient iodine intake in both Greek- and Turkish-Cypriot pregnant women.

Iodine deficiency during pregnancy in Cyprus is of public-health importance, as increasingly evidence points to the fact that even mild-to-moderate iodine deficiency during pregnancy is associated with poorer neurodevelopmental outcomes in the child^(36)^. This is of concern as Cypriot food-based guidelines^(37)^ do not address pregnancy, and there is no official advice on the increment in iodine requirements during pregnancy.

We used a number of methods to assess iodine status – the UIC, UI/Creat and the estimated 24-h urine iodine excretion. We arrived at the same conclusion that the cohort had insufficient iodine status even after using UI/Creat to adjust for urine dilution. A considerable proportion (66 %) of our cohort had a 24-h urine iodine excretion below the value for the EAR in pregnancy (160 µg/24 h). The EAR threshold allows us to describe the proportion of the population affected by iodine deficiency, rather than the overall classification, as provided by the median UIC value^(35)^.

### Iodine supplements and dietary intake

Although 59 % of women reported being supplement users in this cohort, only 32 % took a prenatal supplement that contained iodine; the most popular local supplement did not contain iodine. The proportion of iodine supplement users was similar to women in the UK (28–43 %)^(29,38)^, higher than Norway (18 %)^(8)^, but considerably less than in Iceland (66 %)^(39)^ or Belgium (61 %)^(40)^. Women in our study who used iodine-containing supplements had a higher UI/Creat than those who did not. However, the median UIC and UI/Creat were still below the adequate range, even in those who took iodine supplements. This is similar to findings from other studies in Europe^(29,40,41,42)^ and Australia^(43)^ where iodine-supplement users were still classified as iodine deficient and suggests that gestational supplementation alone may not be enough to guarantee adequate iodine supply (particularly with unplanned pregnancies). We note that five women in this cohort were using brown seaweed (kelp/kombu) supplements, which may provide excess iodine^(44,45)^. Excess iodine intake in pregnancy may increase the risk of maternal thyroid dysfunction^(46)^, especially in cases of chronic low dietary iodine intake. It is important to note that the evidence base for iodine supplementation in mild-to-moderate deficiency is limited^(47)^, and the neurodevelopmental effects of iodine from supplements may be different to that from dietary sources^(48)^. Therefore, it is important to understand the food sources of iodine and ways to ensure adequate iodine intake from the diet, where possible.

The only dietary factor that was a predictor of iodine status in the multivariate model was eggs. This is also in line with other data from studies in Europe that have found eggs to be positively associated with iodine status^(49,50)^. However, we acknowledge that egg intake in this cohort only explained a minor part of the variation in iodine status. We also have no data on the type of egg consumed (e.g. organic/free-range/barn) and whether this would affect egg-iodine concentration, and therefore the relationship with iodine status; we suggest that further research on variation in egg iodine concentration would be useful.

We found no association between iodine status and milk consumption, which is in contrast to results from other European studies in pregnancy^(27,29,39,49,50)^ and is perhaps surprising. Goats’ milk is generally considered to have a higher iodine concentration than that of cows’ milk, but our study may have been underpowered to detect associations given the low numbers of goats’-milk consumers (13 %). Furthermore, there are no data on the iodine concentration of cows’ milk from Cyprus, and therefore, it is not clear whether it is a good source of iodine, as it is in other countries. The iodine concentration of cows’ milk varies across the world^(51)^, probably because of differences in feed and farming practices, and therefore we cannot make assumptions about the iodine concentration of cows’ milk in Cyprus, and whether it would be expected to contribute meaningful amounts of iodine. We therefore recommend that further research is conducted to measure the iodine concentration of milk from Cyprus to fill this gap in knowledge.

It is worth mentioning that many women in our cohort regularly consumed dairy products, which fits with other research in Cyprus showing that dairy foods are consumed by a high proportion of young people^(23)^. Generally, milk and dairy product (e.g. halloumi cheese and yoghurt) consumption and production are relatively high in Cyprus^(52,53)^ and are largely dependent on goats’ and sheeps’ milk, with cows’ milk used less often for the production of dairy products^(54)^. Previous research has found a higher iodine concentration in sheeps’ and goats’ milk than in cows’ milk^(25)^, and so this may explain the fact that in our study there was a slightly stronger (although non-significant) relationship between dairy products (based largely on goats’ milk) and UI/Creat than with milk intake (which was largely cows’ milk).

Fish or seafood intake was not significantly associated with iodine status in our cohort. While we observed a higher UI/Creat in women consuming >1 weekly portion of seafood than those consuming <1, the difference was not significant. This is similar to the findings of other studies of iodine status in pregnant women across Europe^(29)^ but is in contrast to studies that have found fish to be positively associated with iodine status in pregnancy^(39,49,50)^. Our null findings may be explained by small numbers in sub-group analyses within our study and the fact that, as seafood is consumed infrequently, the iodine intake may not be reflected in urine (which reflects the previous 1–2 d of intake^(55)^). Our results may also relate to the type of fish consumed and the fact that there is large variation in fish-iodine content between species which is dependent on seawater and mineral composition^(56)^. Fish intake may be low in pregnancy for a variety of reasons, for example a qualitative study in the UK found that pregnant women reported uncertainties around heavy-metal exposure from fish varieties, difficulties with the taste or smell of fish, and the symptoms of heartburn and morning sickness reducing the likelihood of increasing fish intake^(57)^.

Iodised salt use was low in this cohort, at just 10 %. This is considerably lower than that found in the study in Northern Cyprus^(19)^ (72 %), where there is greater availability in the market. In Europe, there is mandatory salt iodisation in nineteen countries (including Turkey)^(20)^. Overall, concerted action is needed to raise consumer awareness about iodine and iodised salt and to harmonise regulations on salt iodisation^(58)^.

### Strengths and limitations

Ours is the first study in pregnant women from The Republic of Cyprus to examine dietary sources and the effect of iodine supplements on status. However, we acknowledge a number of limitations. First, we are not able to generalise results from one region to reflect the iodine status of the wider Cypriot pregnant population. Second, our study may have been underpowered to detect associations with dietary intake given the relatively small sample size of the subgroups. Third, we did not collect data on other factors that might influence iodine status, such as ethnicity and socio-economic factors (maternal education and income). However, it is likely that our cohort is mostly Greek-Cypriot, as first trimester visits are less frequently attended by other ethnicities^(59)^ and most participants spoke Greek or English. We recruited within a private healthcare setting, but private healthcare in Cyprus is common, inexpensive, and highly accessible. However, in June 2019 (towards the end of our recruitment), the new national General Healthcare System (GeSY) was implemented to provide affordable medical care to Cyprus residents. In theory, this may have meant that women with higher socioeconomic status visited the private rather than the public sector, in which case our findings would reflect a best-case scenario. However, as these changes in healthcare were implemented in Cyprus at the end of our recruitment period, this is unlikely to be a major limitation. Finally, we acknowledge that dietary intake may change throughout pregnancy and that our cross-sectional study is limited by assessment with a single urine sample. This is especially true for the results using the EAR cut-point method, which ideally requires two urine samples, at least in a sub-set of participants^(35)^. We used the 24-h urine iodine excretion value as a proxy for intake, but the assumption that 90 % of iodine is excreted in urine may not be valid in pregnancy as iodine transfer to the fetus, and storage in the placenta^(60)^, may affect the proportion of iodine that is excreted. Furthermore, our assessment of dietary intake (including milk) may have been limited by the inherent limitations of FFQs and this may have influenced the observed (or absent) relationships between iodine status and food groups.

### Further research

We recommend further studies of iodine status in pregnant women in Cyprus, including repeated measures of iodine status throughout pregnancy. A study addressing the effects of mild-to-moderate ID on maternal thyroid function in healthy pregnant women in Cyprus is needed. We also suggest that iodine status in women prior to pregnancy is assessed, especially in view of the high prevalence of infertility in Cyprus and recent research that suggests low iodine may reduce fecundity^(61,62)^.

Given the lack of data on iodine content of foodstuffs available in Cyprus, we suggest a need for analysis of iodine concentration of local milk (cow, goat and sheep) and dairy products. Future studies might also compare thyroid-related biomarkers and thyroid dysfunction between the Turkish-Cypriot (who rely on foods with iodised salt) and the Greek-Cypriot communities, and examine relationships with iodine status and dietary iodine intake.

Finally, as there are no pregnancy-specific recommendations for iodine supplements in Cyprus, and in view of the low iodine status that we have demonstrated, we suggest that Cyprus could be an appropriate setting for an RCT of iodine in pregnancy with follow-up measures of thyroid and neurodevelopmental outcomes in the child. Such an RCT is now challenging to conduct in other countries owing to salt iodisation or recommendations for women to increase their iodine status in pregnancy. However, as 32 % of women in our study were already using iodine-containing supplements, it may still be challenging to recruit women to such a trial.

### Conclusion

This is the first nationwide study demonstrating iodine deficiency in first-trimester pregnant women in the Republic of Cyprus. Our findings add to the evidence base of iodine deficiency in European pregnant women. In view of the low iodine status we observed, we encourage measures to raise awareness, to guide and educate women of childbearing age, especially vegans, about the importance of iodine nutrition. As iodine supplementation at the point of pregnancy may be too late, we suggest that women are given advice to optimise iodine intake and therefore thyroidal iodine stores at least three months prior to conception. Consideration of appropriate iodine fortification policies, as advocated by the Turkish-Cypriot community, may be required to address iodine deficiency.

## References

[1] Zimmermann MB (2009) Iodine deficiency. Endocr Rev 30, 376–408. 19460960 10.1210/er.2009-0011

[2] Bath SC , Steer CD , Golding J , et al. (2013) Effect of inadequate iodine status in UK pregnant women on cognitive outcomes in their children: results from the Avon longitudinal study of parents and children (ALSPAC). Lancet 382, 331–337. 23706508 10.1016/S0140-6736(13)60436-5

[3] Levie D , Korevaar TIM , Bath SC , et al. (2019) Association of maternal iodine status with child IQ: a meta-analysis of individual-participant data. J Clin Endocrinol Metab 104, 5957–5967. 30920622 10.1210/jc.2018-02559 PMC6804415

[4] Zimmermann MB (2009) Iodine deficiency in pregnancy and the effects of maternal iodine supplementation on the offspring: a review. Am J Clin Nutr 89, 668S–672S. 19088150 10.3945/ajcn.2008.26811C

[5] Bougma K , Aboud FE , Harding KB , et al. (2013) Iodine and mental development of children 5 years old and under: a systematic review and meta-analysis. Nutrients 5, 1384–1416. 23609774 10.3390/nu5041384 PMC3705354

[6] Zimmermann MB & Andersson M (2021) Global endocrinology: global perspectives in endocrinology: coverage of iodized salt programs and iodine status in 2020. Eur J Endocrinol 185, R13–R21. 33989173 10.1530/EJE-21-0171 PMC8240726

[7] Hynes KL , Otahal P , Burgess JR , et al. (2017) Reduced educational outcomes persist into adolescence following mild iodine deficiency in utero, despite adequacy in childhood: 15-year follow-up of the gestational iodine cohort investigating auditory processing speed and working memory. Nutrients 9, E1354. 10.3390/nu9121354 PMC5748804 29236073

[8] Markhus MW , Dahl L , Moe V , et al. (2018) Maternal iodine status is associated with offspring language skills in infancy and toddlerhood. Nutrients 10, E1270. 10.3390/nu10091270 PMC6163597 30205599

[9] Murcia M , Espada M , Julvez J , et al. (2017) Iodine intake from supplements and diet during pregnancy and child cognitive and motor development: the INMA mother and child cohort study. J Epidemiol Community Health 72, 216–222. 29279360 10.1136/jech-2017-209830

[10] Costeira MJ , Oliveira P , Santos NC , et al. (2011) Psychomotor development of children from an iodine-deficient region. J Pediatr 159, 447–453. 21492867 10.1016/j.jpeds.2011.02.034

[11] Hynes KL , Otahal P , Hay I , et al. (2013) Mild iodine deficiency during pregnancy is associated with reduced educational outcomes in the offspring: 9-year follow-up of the gestational iodine cohort. J Clin Endocrinol Metab 98, 1954–1962. 23633204 10.1210/jc.2012-4249

[12] Gordon RC , Rose MC , Skeaff SA , et al. (2009) Iodine supplementation improves cognition in mildly iodine-deficient children. Am J Clin Nutr 90, 1264–1271. 19726593 10.3945/ajcn.2009.28145

[13] WHO Secretariat, Andersson M , de Benoist B , et al. (2007) Prevention and control of iodine deficiency in pregnant and lactating women and in children less than 2-years-old: conclusions and recommendations of the technical consultation. Public Health Nutr 10, 1606–1611. 18053287 10.1017/S1368980007361004

[14] WHO, UNICEF & ICCIDD (2007) Assessment of Iodine Deficiency Disorders and Monitoring their Elimination. Geneva: WHO.

[15] Ittermann T , Albrecht D , Arohonka P , et al. (2020) Standardized map of iodine status in Europe. Thyroid 30, 1346–1354. 32460688 10.1089/thy.2019.0353

[16] Bath SC (2017) The challenges of harmonising the iodine supply across Europe. Lancet Diabetes Endocrinol 5, 411–412. 27939394 10.1016/S2213-8587(16)30329-1

[17] Iodine Global Network (2021) Global Scorecard of Iodine Nutrition in 2021. https://www.ign.org/cm_data/IGN_Global_Scorecard_2021_7_May_2021.pdf (accessed October 2021).

[18] Erdogan G , Sav H & Erdogan MF (2000) Iodine status and thyroid volumes of school age children from Northern Cyprus. J Endocrinol Invest 23, 74–77. 10800758 10.1007/BF03343682

[19] Mousa U , Sav H , Koseoglulari O , et al. (2018) Iodine status of pregnant women in Northern Cyprus. Balkan Med J 35, 449–450. 30203779 10.4274/balkanmedj.2018.0679 PMC6251378

[20] Global Fortification Data Exchange (2019) Map: Fortification Legislation. https://fortificationdata.org/interactive-map-fortification-legislation/# (accessed April 2020).

[21] Lazarou C , Panagiotakos DB & Matalas AL (2009) Level of adherence to the Mediterranean diet among children from Cyprus: the CYKIDS study. Public Health Nutr 12, 991–1000. 18752695 10.1017/S1368980008003431

[22] Kyriacou A , Evans JM , Economides N , et al. (2015) Adherence to the Mediterranean diet by the Greek and Cypriot population: a systematic review. Eur J Public Health 25, 1012–1018. 26130797 10.1093/eurpub/ckv124

[23] Hadjimbei E , Botsaris G , Gekas V , et al. (2016) Adherence to the Mediterranean diet and lifestyle characteristics of university students in Cyprus: a cross-sectional survey. J Nutr Metab 2016, 2742841. 27293883 10.1155/2016/2742841 PMC4884852

[24] Food Standards Agency (2008) Retail Survey of Iodine in UK Produced Dairy Foods. FSIS 02/08. http://tna.europarchive.org/20140306205048/http://www.food.gov.uk/science/research/surveillance/fsisbranch2008/fsis0208 (accessed April 2015).

[25] Stergiadis S , Nørskov NP , Purup S , et al. (2019) Comparative nutrient profiling of retail goat and cow milk. Nutrients 11, 2282. 31554167 10.3390/nu11102282 PMC6835441

[26] Andersen S , Karmisholt J , Pedersen KM , et al. (2008) Reliability of studies of iodine intake and recommendations for number of samples in groups and in individuals. Br J Nutr 99, 813–818. 17961291 10.1017/S0007114507842292

[27] Bath SC , Furmidge-Owen VL , Redman CW , et al. (2015) Gestational changes in iodine status in a cohort study of pregnant women from the United Kingdom: season as an effect modifier. Am J Clin Nutr 101, 1180–1187. 25948667 10.3945/ajcn.114.105536 PMC4441812

[28] Bingham SA , Welch AA , McTaggart A , et al. (2001) Nutritional methods in the European prospective investigation of cancer in Norfolk. Public Health Nutr 4, 847–858. 11415493 10.1079/phn2000102

[29] Bath SC , Walter A , Taylor A , et al. (2014) Iodine deficiency in pregnant women living in the South East of the UK: the influence of diet and nutritional supplements on iodine status. Br J Nutr 111, 1622–1631. 24398008 10.1017/S0007114513004030 PMC4346198

[30] Bath SC , Hill S , Infante HG , et al. (2017) Iodine concentration of milk-alternative drinks available in the UK in comparison with cows’ milk. Br J Nutr 118, 525–532. 28946925 10.1017/S0007114517002136 PMC5650045

[31] UNICEF (2018) Guidance on the Monitoring of Salt Iodization Programmes and Determination of Population Iodine Status. https://www.unicef.org/nutrition/files/Monitoring-of-Salt-Iodization.pdf (accessed June 2018).

[32] Knudsen N , Christiansen E , Brandt-Christensen M , et al. (2000) Age- and sex-adjusted iodine/creatinine ratio. A new standard in epidemiological surveys? Evaluation of three different estimates of iodine excretion based on casual urine samples and comparison to 24 h values. Eur J Clin Nutr 54, 361–363. 10745289 10.1038/sj.ejcn.1600935

[33] Konig F , Andersson M , Hotz K , et al. (2011) Ten repeat collections for urinary iodine from spot samples or 24-h samples are needed to reliably estimate individual iodine status in women. J Nutr 141, 2049–2054. 21918061 10.3945/jn.111.144071

[34] Vejbjerg P , Knudsen N , Perrild H , et al. (2009) Estimation of iodine intake from various urinary iodine measurements in population studies. Thyroid 19, 1281–1286. 19888863 10.1089/thy.2009.0094

[35] Zimmermann MB & Andersson M (2012) Assessment of iodine nutrition in populations: past, present, and future. Nutr Rev 70, 553–570. 23035804 10.1111/j.1753-4887.2012.00528.x

[36] Bath SC (2019) The effect of iodine deficiency during pregnancy on child development. Proc Nutr Soc 78, 150–160. 30642416 10.1017/S0029665118002835

[37] Ministry of Health. (2007) Nutrition and Exercise Guidelines for Adults in Cyprus. http://www.nut.uoa.gr/dietaryENG.html (accessed May 2021).

[38] Knight BA , Shields BM , He X , et al. (2016) Iodine deficiency amongst pregnant women in South-West England. Clin Endocrinol 86, 451–455. 10.1111/cen.13268 27805280

[39] Gunnarsdottir I , Gustavsdottir AG , Steingrimsdottir L , et al. (2013) Iodine status of pregnant women in a population changing from high to lower fish and milk consumption. Public Health Nutr 16, 325–329. 22607718 10.1017/S1368980012001358 PMC10271330

[40] Vandevijvere S , Amsalkhir S , Mourri AB , et al. (2013) Iodine deficiency among Belgian pregnant women not fully corrected by iodine-containing multivitamins: a national cross-sectional survey. Br J Nutr 109, 2276–2284. 23084115 10.1017/S0007114512004473

[41] Lindorfer H , Krebs M , Kautzky-Willer A , et al. (2015) Iodine deficiency in pregnant women in Austria. Eur J Clin Nutr 69, 349–354. 25491497 10.1038/ejcn.2014.253

[42] Alvarez-Pedrerol M , Ribas-Fito N , Garcia-Esteban R , et al. (2010) Iodine sources and iodine levels in pregnant women from an area without known iodine deficiency. Clin Endocrinol 72, 81–86. 10.1111/j.1365-2265.2009.03588.x 19508607

[43] Clifton VL , Hodyl NA , Fogarty PA , et al. (2013) The impact of iodine supplementation and bread fortification on urinary iodine concentrations in a mildly iodine deficient population of pregnant women in South Australia. Nutr J 12, 32. 23497409 10.1186/1475-2891-12-32 PMC3621546

[44] Leung AM , Pearce EN & Braverman LE (2009) Iodine content of prenatal multivitamins in the United States. N Engl J Med 360, 939–940. 19246372 10.1056/NEJMc0807851

[45] Zimmermann M & Delange F (2004) Iodine supplementation of pregnant women in Europe: a review and recommendations. Eur J Clin Nutr 58, 979–984. 15220938 10.1038/sj.ejcn.1601933

[46] Crawford BA , Cowell CT , Emder PJ , et al. (2010) Iodine toxicity from soy milk and seaweed ingestion is associated with serious thyroid dysfunction. Med J Aust 193, 413–415. 20919974 10.5694/j.1326-5377.2010.tb03972.x

[47] Dineva M , Fishpool H , Rayman MP , et al. (2020) Systematic review and meta-analysis of the effects of iodine supplementation on thyroid function and child neurodevelopment in mildly-to-moderately iodine-deficient pregnant women. Am J Clin Nutr 112, 389–412. 32320029 10.1093/ajcn/nqaa071

[48] Abel MH , Caspersen IH , Meltzer HM , et al. (2017) Suboptimal maternal iodine intake is associated with impaired child neurodevelopment at 3 years of age in the Norwegian mother and child cohort study. J Nutr 147, 1314–1324. 28515161 10.3945/jn.117.250456

[49] Brantsaeter AL , Abel MH , Haugen M , et al. (2013) Risk of suboptimal iodine intake in pregnant Norwegian women. Nutrients 5, 424–440. 23389302 10.3390/nu5020424 PMC3635203

[50] Dineva M , Rayman MP , Levie D , et al. (2019) Similarities and differences of dietary and other determinants of iodine status in pregnant women from three European birth cohorts. Eur J Nutr 59, 371–387. 30734058 10.1007/s00394-019-01913-w

[51] van der Reijden OL , Zimmermann MB & Galetti V (2017) Iodine in dairy milk: sources, concentrations and importance to human health. Best Pract Res Clin Endocrinol Metab 31, 385–395. 29221567 10.1016/j.beem.2017.10.004

[52] CyStat. (2016) Agricultural Statistics, 1960–2016. https://www.mof.gov.cy/mof/cystat/statistics.nsf/agriculture_51main_en/agriculture_51main_en?OpenForm&sub=1&sel=2 (accessed October 2021).

[53] CyStat. (2019) Cyprus in Figures 2019. http://www.cystat.gov.cy/mof/cystat/statistics.nsf/All/C91603BD82050327C22582030022C7F2/$file/CYPRUS_IN_FIGURES-2019-EN-201219.pdf?OpenElement (accessed September 2019).

[54] Department of Agriculture (2019) Ministry of Agriculture, Rural Development and Environment – Livestock Production and Nutrition Section. Sheep and Goats/Animal Nutrition Section. http://www.moa.gov.cy/moa/da/da.nsf/page17_en/page17_en?OpenDocument (accessed October 2021).

[55] Rasmussen LB , Ovesen L & Christiansen E (1999) Day-to-day and within-day variation in urinary iodine excretion. Eur J Clin Nutr 53, 401–407. 10369497 10.1038/sj.ejcn.1600762

[56] Sprague M , Chau TC & Givens DI (2021) Iodine content of wild and farmed seafood and its estimated contribution to UK dietary iodine intake. Nutrients 14, 195. 35011067 10.3390/nu14010195 PMC8747335

[57] Bouga M , Lean MEJ & Combet E (2018) Iodine and pregnancy-a qualitative study focusing on dietary guidance and information. Nutrients 10, 408. 29587423 10.3390/nu10040408 PMC5946193

[58] Euthyroid Consortium (2018) The Krakow declaration on iodine: tasks and responsibilities for prevention programs targeting iodine deficiency disorders. Eur Thyroid J 7, 201–204. 30283738 10.1159/000490143 PMC6140595

[59] CyStat. (2007) Perinatal Health Survey 2007. https://www.mof.gov.cy/mof/cystat/statistics.nsf/All/5D31FBE47D499BB4C22577EA00306C6A/$file/PERINATAL_HEALTH_SURVEY_2007–291110.pdf?OpenElement (accessed October 2021).

[60] Neven KY , Marien CBD , Janssen BG , et al. (2020) Variability of iodine concentrations in the human placenta. Sci Rep 10, 161. 31932629 10.1038/s41598-019-56775-3 PMC6957482

[61] Abel MH , Caspersen IH , Sengpiel V , et al. (2020) Insufficient maternal iodine intake is associated with subfecundity, reduced foetal growth, and adverse pregnancy outcomes in the Norwegian mother, father and child cohort study. BMC Med 18, 211. 32778101 10.1186/s12916-020-01676-w PMC7418397

[62] Mills JL , Buck Louis GM , Kannan K , et al. (2018) Delayed conception in women with low-urinary iodine concentrations: a population-based prospective cohort study. Hum Reprod 33, 426–433. 29340704 10.1093/humrep/dex379 PMC6454505

